# Acquired Factor VIII Inhibitors: Three Cases

**DOI:** 10.4274/tjh.2012.0009

**Published:** 2013-03-05

**Authors:** Tay Za Kyaw, S. Jayaranee, Ping Chong Bee, Edmund Fui Min Chin

**Affiliations:** 1 Malaya University Faculty of Medicine, Department of Pathology, Kuala Lumpur, Malaysia; 2 Malaya University Faculty of Medicine, Department of Medicine, Kuala Lumpur, Malaysia

**Keywords:** Acquired hemophilia, FVIII inhibitors, Hemarthrosis, Postpartum inhibitors

## Abstract

Acquired hemophilia A is a rare, but devastating bleeding disorder caused by spontaneous development of autoantibodies directed against coagulation factor VIII. In 40%-50% of patients it is associated with such conditions as the postpartum period, malignancy, use of medications, and autoimmune diseases; however, its cause is unknown in most cases. Acquired hemophilia A should be suspected in patients that present with a coagulation abnormality, and a negative personal and family history of bleeding. Herein we report 3 patients with acquired hemophilia A that had different underlying pathologies, clinical presentations, and therapeutic responses. Factor VIII inhibitor formation in case 1 occurred 6 months after giving birth; underlying disorders were not identified in cases 2 or 3. The bleeding phenotype in these patients’ ranged from no bleeding tendency with isolated prolongation of APTT (activated partial thromboplastin time) to severe intramuscular hematoma and hemarthrosis necessitating recombinant activated factor VII infusion and blood components transfusion. Variable responses to immunosuppressive treatment were also observed.

**Conflict of interest:**None declared.

## Abstract (tr)

Edinsel hemofili A, faktör VIII’e karşı kendiliğinden gelişen otoantikorlar nedeniyle oluşan nadir ancak yıkıcı bir kanama hastalığıdır. Olguların %40-50’de doğum sonrası dönem, malignansi, ilaç kullanımı ve otoimmun hastalıklar bu duruma neden olurken, diğer olgularda neden çoğu zaman bulunamaz. Kanamaya yönelik kişisel ve ailesel öyküsü bulunmayan ve koagulasyon bozukluğu olan olgularda edinsel hemofili A dan mutlaka şüphelenmeliyiz. Bu yazıda altta yatan farklı patolojileri, klinik başvuruları ve tedavi cevapları olan 3 edinsel hemofili A olgularını sunduk. İlk olguda faktör VIII inhibitör oluşumu doğumdan 6 ay sonra gelişirken ikinci ve üçüncü olguda altta yatan neden bulunamamıştır. Bu olguların kanama fenotipi hiçbir kanama eğilimi olmadan izole aPTT (aktive parsiyel tromboplastin zamanı) uzaması ya da kan ürünü transfüzyonu ve rekombinant aktive faktör VII infüzyonu gerektiren hemartroz ve ciddi kas içi kanaması şeklinde değişmekteydi.Immunsupresif tedaviye değişken yanıtları da gözlenmiştir.

## INTRODUCTION

Acquired hemophilia A (AHA) is a rare bleeding disorder caused by spontaneous development of autoantibodies directed against coagulation factor VIII (FVIII). It has an incidence of approximately 1.5 cases/million/year [1]. The etiology of AHA is obscure in the majority of cases. In about 40%-50% of patients AHA is associated with such underlying conditions as the postpartum period, malignancy, use of medications, and autoimmune diseases, including rheumatoid arthritis and systemic lupus erythematosus (SLE) [2]. AHA is more common in adults and is equally distributed between the sexes [3]. 

The diagnosis of AHA is often difficult because of the lack of personal or family history of bleeding [4]. The bleeding pattern in AHA differs from that observed in congenital hemophilia. Hemarthrosis, a typical bleeding manifestation of congenital FVIII deficiency, is uncommon in AHA. The majority of patients with AHA present with bleeding into the skin, muscles, soft tissues, and mucous membranes (e.g. epistaxis, gastrointestinal and urological bleeding, and retroperitoneal hematomas) [4]. Severe or life-threatening bleeding occurs in >80% of patients and typically occurs early in the course of the disease [1,5]. Mortality due to AHA is reported to be 9%-22% [1,5]. Herein we report 3 patients with AHA that had different underlying pathologies, clinical presentations, and therapeutic responses.

## CASE REPORT

**Case 1**

Written informed consent was obtained from the patients.

In September 1998 a 28-year-old Chinese female was referred to our center for further investigation of incidentally observed prolonged APTT (activated partial thromboplastin time) 6 months post delivery of her first child. Family and personal history of bleeding diathesis were negative. Laboratory investigation showed an APTT of 69.6 s (reference range: 28.5-38 s), which was not corrected via mixing with an equal volume of normal plasma. Her FVIII activity was 2% (reference range: 50%-150%) and her FVIII inhibitor level was 512 Bethesda units (BU). Prothrombin time, thrombin time, and full blood count (FBC) were normal. Lupus anticoagulant (LA), anti-cardiolipin antibody (ACA), and hepatitis screening results were negative. Connective tissue disease (CTD) screening showed a normal level of anti-DNA antibody and a significantly elevated anti-nuclear factor (ANF) titer (1:1280); however, the patient’s clinical features did not indicate CTD.

Based on the high-titer FVIII inhibitors, prednisolone and azathioprine were initiated. Prednisolone was gradually tapered off and stopped after about 1 year, at which time the FVIII inhibitor level was 115 BU. Azathioprine was discontinued in September 2001 following observation of a marked reduction in FVIII inhibitor (2 BU) and improved FVIII activity (23%). Two years later FVIII inhibitors disappeared spontaneously and FVIII activity increased to 47%. When the patient returned to our hospital in 2007 for the antenatal care during her second pregnancy, her routine coagulation screening was normal and her FVIII level was 120%. CTD screening was not repeated due to the absence of clinical signs and symptoms of CTD. 

**Case 2**

A 23-year-old single Chinese female presented to our center in late February 2009 with complaints of easy bruising and bilateral leg swelling. History and physical examination were normal. Doppler ultrasound of her lower legs showed no evidence of thrombosis. Coagulation profile showed isolated prolongation of APTT (66.5 s), which was not corrected via mixing with normal plasma. 

FVIII activity was markedly reduced (2.8%), with a high FVIII inhibitor titer (61 BU). FIX, FXI, and FXII activity were low; however, a repeat of FIX, FXI, and FXII assays after serial dilution of the patient’s plasma showed activity of 94%, 67%, and 50%, respectively. LA was negative. Other coagulation test results were normal. FBC, thyroid function test, and CTD screening, which included C3, C4, ANF, ACA, and rheumatoid factor, findings were normal. HBsAg and anti-Hepatitis C virus antibodies were negative. Thoracic and abdominal computerized tomography (CT) findings were normal. 

The patient was started on prednisolone 60 mg/d in March 2009 and 1 month later azathioprine 50 mg/d was added. After a few weeks of treatment the swelling in both legs completely resolved. In October 2009 prednisolone was discontinued, and the FVIII inhibitor level was undetectable and FVIII activity was 24.6%. Then, 6 months later the patient’s FVIII activity was normal (52.4%). Azathioprine was stopped in March 2011 and at the time this manuscript was prepared the patient was doing well. Her APTT at the last follow-up was normal. 

**Figure 1:**
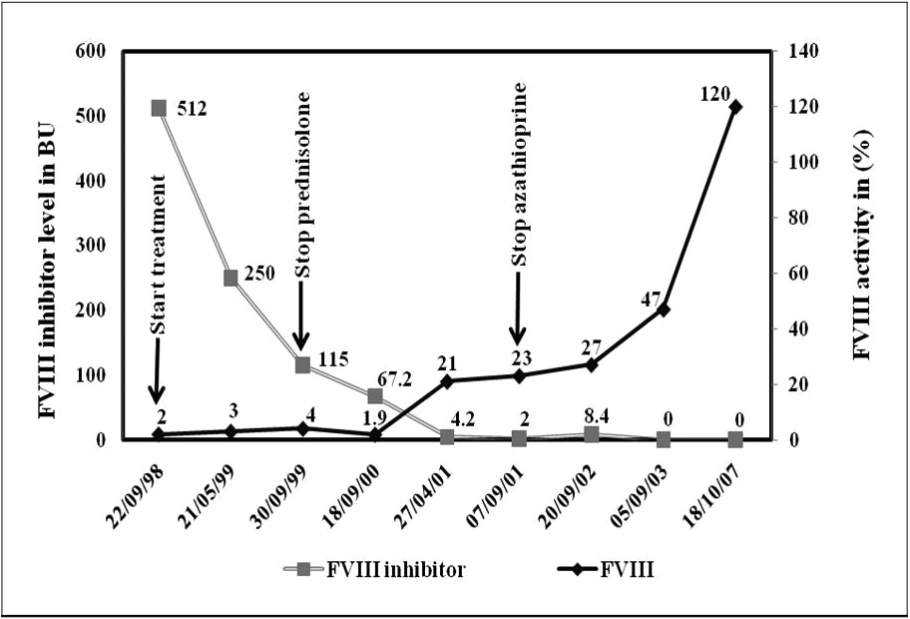
Factor VIII activity and FVIII inhibitor levels during and after the immunosuppressive therapy in the first patient

**Figure 2:**
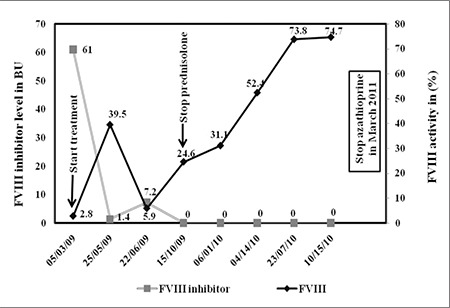
Factor VIII activity and FVIII inhibitor levels during and after the immunosuppressive therapy in the second patient

FVIII activity and FVIII inhibitor levels in cases 1 and 2-during and after treatment with immunosuppressive agents-are shown in Figures 1 and 2, respectively. 

**Case 3**

A 71-year-old female presented to our hospital in December 2010 with acute onset of right knee pain and swelling associated with easy bruising. She had been taking amlodipine and simvastatin for hypertension and hypercholesterolemia, respectively. Ten years earlier she was diagnosed with cervical carcinoma and underwent hysterectomy followed by chemotherapy. 

**Figure 3:**
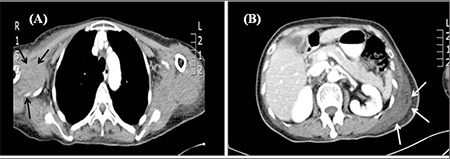
(A) CT scan image demonstrating a subcutaneous haematoma in the right axilla region impinging on the surrouding vessels (black arrows). (B) CT scan image demostrating an intramuscular haematoma in the left lattissimus dorsi muscle (white arrows)

Physical examination showed right knee swelling and tenderness associated with limited movement due to the underlying hemarthrosis. Bruising was present on the patient’s forearms. Whole-body CT showed a soft tissue hematoma in the right axilla that extended to the right shoulder girdle and medial aspect of the right upper arm, and an intramuscular hematoma in the left latissimus dorsi that extended to the lumbar region. No other abnormality was detected (Figure 3). Coagulation profile showed isolated prolongation of APTT (106.3 s), which was not corrected via mixing with normal plasma. The FVIII level was markedly reduced (0.9%), with an FVIII inhibitor level of 35 BU. FBC at admission was as follows: hemoglobin: 9.9 g/dL, white cell count: 9.4 × 10^9^/L; platelet count: 359 × 10^9^/L. CTD screening results were normal. LA was negative. Tumor markers (CEA, CA125, and CA19-9) were within the normal range. 

During this admission she developed new intramuscular hematomas and hemarthrosis, hemoglobin dropped to 5.6 g/dL, and she was given recombinant activated factor VII (rFVIIa) 0.9 µg/kg as a single dose together with blood components; i.v. methylprednisolone 500 mg/d was started simultaneously and was changed to oral prednisolone 40 mg/d after 3 d. Cyclophosphamide 100 mg/d i.v. was added for 3 d and was later switched to oral cyclophosphamide 50 mg/d during hospitalization. Although there was significant clinical improvement, FVIII activity before discharge was only 3.9% and the FVIII inhibitor concentration was 54.4 BU. She was discharged in January 2011 and prescribed prednisolone 40 mg/d. Her APTT test results have been normal since April 2011. Her FVIII activity in June 2011 was 58%, with undetectable FVIII inhibitors. She is currently taking prednisolone 5 mg/d and is asymptomatic. 

## DISCUSSION

Herein we described 3 AHA cases with different clinical presentations, underlying pathologies, and therapeutic responses. The bleeding phenotype in these patients ranged from no bleeding tendency with isolated prolongation of APTT, to severe intramuscular hematoma and hemarthrosis necessitating rVIIa infusion and blood components transfusion. The occurrence of hemarthrosis in case 3 is unusual, but there are other reports of a similar presentation [6]. 

In case 1 FVIII inhibitor formation was probably a result of the postpartum state. Postpartum FVIII inhibitors usually develop 1-4 months post delivery, but may occur as late as 1 year [7]. FVIII inhibitor formation can occur following any pregnancy, but is observed more often after the first birth, as in case 1 [7]. The majority of patients with postpartum AHA have low-titer FVIII inhibitors, which tends to disappear spontaneously after a median period of 30 months [4,8]; however, high-titer postpartum FVIII inhibitors may persist for years despite immunosuppressive therapy and may precede the development of an overt autoimmune disease [4]. Case 1 presented with high-titer FVIII inhibitors that was resistant to immunosuppressants. Her CTD screening at presentation showed a high ANF titer. The possibility of AHA as a preceding event of CTD cannot be completely excluded in this patient. Although no feature of CTD was observed during the 5-year follow-up of AHA or during the follow-up of her second pregnancy, it is important that we continue to monitor for features of CTD in case 1. 

Underlying disorders such as CTD, malignancy, hepatitis, and dermatological and respiratory diseases were not detected in cases 2 and 3. Although case 3 has been taking anti-hypertensive and anti-lipemic medications, none have been reported to cause AHA. Additionally, there was no evidence of malignancy in case 3. Idiopathic AHA often occurs in elderly individuals and usually presents with life-threatening bleeding, as in case 3 [4]. All possible underlying causes must be carefully investigated and excluded before making a diagnosis of idiopathic AHA. Patients with idiopathic AHA should also undergo long-term follow-up, which is particularly true for those aged less than 40 years in whom the occurrence of idiopathic AHA is uncommon [1]. The importance of long-term follow-up is highlighted in a report by Hsieh et al. [9] of a 38-year-old female that initially presented with AHA, and then developed SLE 7 years later. 

The management of AHA is two fold: (1) to control bleeding and (2) to eradicate FVIII inhibitors. Hemostatic control is necessary in patients with active severe bleeding, irrespective of inhibitor titer [10]. Several therapeutic approaches have been utilized for the control of bleeding, including bypassing agents, such as rVIIa and activated prothrombin complex concentrate (aPCC), porcine FVIII, human or recombinant FVIII, and desmopressin (DDAVP) [4]. Plasma-derived porcine FVIII concentrate was successfully used in the past to manage AHA, in particular because FVIII autoantibodies often have low cross-reactivity with porcine FVIII; however, it is no longer commercially available, but a recombinant porcine B-domain-depleted FVIII molecule is under development [10]. The choice of treatment agent depends on the severity of bleeding and the FVIII inhibitor titer [2]. Thus, while clinically mild cases of AHA with low-titer inhibitors can be managed successfully using DDAVP or FVIII concentrates, patients with high-titer FVIII inhibitors and severe bleeding benefit from bypassing agents [8]. 

Currently, bypassing agents rVIIa and aPCC are the most frequently used first-line treatments; both of these bypassing agents have been proven effective in the treatment of AHA [11]. A large retrospective study by Goudemand and the French FEIBA Study Group reported that aPCC had good hemostatic efficacy in 89% of bleeding episodes [12]. The first large-scale study on the use of rVIIa in patients with AHA was a retrospective analysis of 38 patients by Hay et al. [13]. The 38 patients were treated for 78 bleeding episodes using rVIIa, and 100% of patients had a good response when rVIIa was used as the first-line treatment and in 75% of patients when it was used as salvage therapy. Case 3 in the present study had severe bleeding episodes with significantly reduced hemoglobin that required use of rVIIa and blood components, and marked clinical improvement was observed following rVIIa infusion. In case 2 although swelling in the legs was probably caused by intramuscular hemorrhaging, the patient was clinically stable with a normal hemoglobin level; thus, hemostatic control was not required.

The second aim of AHA management is eradication of FVIII autoantibodies. Although spontaneous resolution of FVIII inhibitors may occur in up to 30% of patients [14], the occurrence is unpredictable and patients remain at risk of severe bleeding if the inhibitors persist [15]; therefore, immunosuppressive therapy for eradication of inhibitors is recommended in all AHA patients [10]. In contrast to AHA, immunosuppressive therapy has had limited success in eradicating FVIII alloantibodies in congenital hemophilia patients [16]. In severe congenital hemophilia A patients with FVIII inhibitors, immune tolerance induction (ITI) therapy using regular applications of FVIII for a certain period is the only proven strategy for eradication of FVIII inhibitors and induction of FVIII-specific immune tolerance [17]. The most widely used ITI strategies [Bonn protocol and van Creveld protocol] require regular infusion of FVIII products without using immunosuppressive agents [17]. Patients with AHA FVIII autoantibodies exhibit rapid and non-linear inactivation of FVIII antigens (type 2 kinetics) [3]; therefore, it is difficult to saturate these autoantibodies by adding FVIII antigens. High-dose FVIII infusion therapy used to eradicate inhibitors in congenital hemophilia was unsuccessful in AHA, particularly in high-titer patients [18]. 

The most successful immunosuppressive regimens for the management of AHA are corticosteroids alone or in combination with cyclophosphamide [2]. A non-randomized study by the United Kingdom Hemophilia Center Doctors’ Organization [1] reported that there wasn’t a significant difference between groups treated with steroids alone or in combination with cytotoxic agents. Nonetheless, a randomized prospective trial that included 31 AHA patients [15] reported that 50% of steroid-resistant patients responded to a cyclophosphamide-containing regimen. Combination therapies, such as steroids with azathioprine or with other cytotoxic agents, have also been shown to be effective [10]. In non-responders, alternative approaches have been proposed in particular, rituximab. Rituximab is a monoclonal antibody (against the pan B-cell antigen CD20) that induces rapid in vivo depletion of normal B-lymphocytes [19]. Although earlier reports of this agent’s effectiveness were based primarily on lymphomas and other autoimmune diseases, recent studies have reported promising results in terms of eradicating FVIII inhibitors at a common dose of 375 mg/m^2^/week for up to 4 weeks [10]. Rituximab may also be useful as a first-line therapy when chemotherapeutic agents are contraindicated [10].

Case 1 in the present study was given immunosuppressants immediately because of high-titer FVIII inhibitors, even though she was asymptomatic. Her FVIII inhibitors persisted despite receiving immunosuppressive treatment for 3 years. The inhibitors disappeared spontaneously 2 years later. The effect of immunosuppressive therapy on the natural history of FVIII inhibitors in postpartum women is unclear. A retrospective analysis of 51 postpartum FVIII inhibitor patients reported that steroid treatment was no better than no treatment. Immunosuppression did not induce complete remission, but may have slightly reduced the time to complete remission [20]. In contrast to case 1, cases 2 and 3 had good responses to immunosuppressants, and cases 1 and 2 received similar treatment. FVIII inhibitors in case 2 disappeared 6 months after the start of treatment. In case 3 cyclophosphamide was given during hospitalization; however, she was discharged on prednisolone only. Her FVIII level was normal and FVIII inhibitors were undetectable 5 months after initiating prednisolone treatment. Case 3 appeared to have achieved remission with prednisolone alone; however, cyclophosphamide probably contributed to shortening the time to remission. 

In conclusion, AHA is a heterogeneous condition in terms of clinical characteristics, pathogenicity, and therapeutic response, as in the presented cases. Although it is generally recommended that all AHA patients receive immunosuppressants to eradicate FVIII inhibitors immediately following diagnosis, so as to avoid the risk of fatal hemorrhage, the optimal therapeutic strategy has not yet been defined [10]. Large, multinational collaborative randomized controlled trials are required for better assessment of treatment regimes in these patients with a rare, but devastating disorder. 

**Conflict of Interest Statement**

The authors have no conflicts of interest relevant to the materials presented in this manuscript.
